# Displayed Depression Symptoms on Facebook at Two Time Points: Content Analysis

**DOI:** 10.2196/20179

**Published:** 2021-05-31

**Authors:** Megan A Moreno, Quintin Gaus, Megan Wilt, Alina Arseniev-Koehler, Adrienne Ton, Molly Adrian, Ann VanderStoep

**Affiliations:** 1 Department of Pediatrics University of Wisconsin-Madison Madison, WI United States; 2 Department of Nursing St Catherine University Minneapolis, MN United States; 3 Department of Sociology University of California, Los Angeles Los Angeles, CA United States; 4 Department of Nursing Columbia University New York, NY United States; 5 Department of Psychiatry University of Washington Seattle, WA United States

**Keywords:** adolescents, content analysis, depression, Facebook, social media

## Abstract

**Background:**

Depression is a prevalent and problematic mental disorder that often has an onset in adolescence. Previous studies have illustrated that depression disclosures on social media are common and may be linked to an individual’s experiences of depression. However, most studies have examined depression displays on social media at a single time point.

**Objective:**

This study aims to investigate displayed depression symptoms on Facebook at 2 developmental time points based on symptom type and gender.

**Methods:**

Participants were recruited from an ongoing longitudinal cohort study. The content analysis of text-based Facebook data over 1 year was conducted at 2 time points: time 1 (adolescence; age 17-18 years) and time 2 (young adulthood; ages 20-22 years). Diagnostic criteria for depression were applied to each post to identify the displayed depression symptoms. Data were extracted verbatim. The analysis included nonparametric tests for comparisons.

**Results:**

A total of 78 participants’ Facebook profiles were examined, of which 40 (51%) were male. At time 1, 62% (48/78) of the adolescents had a Facebook profile, and 54% (26/78) displayed depression symptom references with an average of 9.4 (SD 3.1) references and 3.3 (SD 2.3) symptom types. Of the 78 participants, 15 (19%) females and 12 (15%) males displayed depression symptom references; these prevalence estimates were not significantly different by gender (*P*=.59). At time 2, 35 young adults displayed symptoms of depression with an average of 4.6 (SD 2.3) references and 2.4 (SD 1.3) symptom types. There were no differences in the prevalence of symptoms of depression displayed between males (n=19) and females (n=16; *P*=.63).

**Conclusions:**

This content analysis study within an ongoing cohort study illustrates the differences in depression displays on Facebook by developmental stage and symptom. This study contributes to a growing body of literature by showing that using social media to observe and understand depression during the emerging adult developmental period may be a valuable approach.

## Introduction

### Background

Depression often has an onset during adolescence and young adulthood [1-3]. Social networking sites (SNSs) may present new opportunities to understand emerging adults’ experiences of depression, as over 90% of youth and 75% of adults use one or more SNS [4,5]. Previous work has found that displayed depression symptoms on SNSs are common; a study of college students found that these displays were present on up to a quarter of participants’ profiles [6]. Furthermore, displayed depression symptoms on SNSs were positively associated with self-reported depression symptoms using clinical screening tools [7]. Given the nearly ubiquitous use of SNSs among adolescents and young adults, the disclosure of depression on SNSs may present an innovative opportunity to evaluate how depression displays on social media may change during the developmental transitions of emerging adulthood. Thus, SNSs may allow researchers to understand unique aspects of the depression experience during adolescence and young adulthood, such as gender differences or changes in the types of symptoms displayed over time. Research approaches incorporating SNSs may enhance our understanding of symptom burden, symptoms over time, and differences by developmental stage and gender.

### Depression During the Emerging Adult Years

The emergence of depression is a major health concern during adolescence; the prevalence of depression increases between the ages of 10 and 18 years [1-3]. The most common form of depression in adolescents and young adults is major depressive disorder, with a yearly incidence of approximately 8% [8,9]. An additional 22% of adolescents and young adults have *subdiagnostic* levels of depression symptoms, which is an important and understudied group [10].

Patients with a major depression diagnosis and those with *subdiagnostic* levels of depression symptoms experience impaired functioning and morbidity. It is well established that depression is associated with numerous negative health and social outcomes. These outcomes can include poor academic achievement, increased rates of substance use, comorbid psychiatric conditions, and, most severely, suicide [11-15].

### Depression and Gender

Gender is another critical factor in the depression experience; previous work suggests that beginning in adolescence, females have higher rates of depression compared with males [16-18]. It has been proposed that gender differences reported in the literature may reflect differences in depression prevalence and depression symptom types or differences in diagnosis and treatment by providers [19]. A recent meta-analysis found that gender differences in depression diagnoses were seen at the young age of 12 years, peaked in adolescence, and declined in adulthood [20].

### A New Lens to Understand Depression Experiences: SNSs

SNSs may present new opportunities to investigate depression, particularly among adolescents and young adults. The vast majority of adolescents and young adults use an SNS, such as Instagram, Snapchat, or Facebook [5,21]. SNSs allow users to build a personal profile, communicate with others, and build a social network. Thus, SNSs provide unique tapestry for curating personal content and sharing with viewers.

Through content displayed on an SNS profile, profile owners may provide researchers insight into experiences that are not always apparent in their life offline. SNSs, such as Facebook, allow individuals to create personal profiles that are public representations of themselves. Facebook users have the ability to change their web-based identity on a daily or even minute-to-minute basis. These disclosures may include references to depression symptoms in the form of *status updates*, that is, personally written text describing the profile owner’s current state of mind. Thus, SNSs allow users to capture their lived feelings, behaviors, and experiences at any time or place.

Examining content on an SNS as a representation of one’s self has theoretical support. Even before the advent of using social media as a venue for personal expression, research has shown that human-computer interaction use fostered self-disclosure and uninhibited personal expression [22,23]. Social media may foster the self-expression of depression symptoms through their unique affordances. Affordances are typically described as properties of artifacts that can be recognized by users and contribute to their function [24]. An affordance approach can be useful because it provides a way to identify functionalities that are similar and different across these platforms [25-28]. Social media affordances can include social affordances, cognitive affordances, or functional affordances. Social affordances provided by social media may enhance the likelihood of disclosures of depression symptoms in social media venues and include a sense of belonging to a group, such as a group focused on a particular interest, experience, social group, or religion [29]. This sense of belonging may lead to social media being a safe space in which depression disclosures can occur. Another social affordance is its capacity to promote network-informed associations, such as when Facebook suggests friends for a user based on their friends’ friends. This allows users to see how friends are connected to other people and their interests [30]. This feature may again contribute to the sense that Facebook is a safe and connected space for disclosure. These affordances may inform mechanisms by determining which types of communication are common on particular platforms.

Affordances are also a useful lens through which to view social media platforms. As platforms can change in popularity or features within an individual platform change, researchers can focus on underlying affordances to interpret observations and predict whether similar observations would be seen on platforms with similar affordances [31].

### Evidence Supporting Depression Symptom Disclosures on SNSs

Several areas of previous research support the investigation of depression using SNSs. First, previous work illustrates that from the inception of social media, adolescents have used SNSs to disclose personal information and seek support. One study from the early period of social media found that adolescents reported that they often disclosed more about themselves on SNSs than they did in person [32]. Adolescents with depression have also described using SNSs to express themselves and seek social support [33]. Peers may respond to these disclosures with social support. Previous work supports that peers often view SNS depression symptom disclosures as a *cry for help* and respond by providing support on the web and offline [34].

Second, depression symptom displays are common across SNSs. Previous studies have described depression symptom displays across various SNSs, including Facebook, Twitter, and Tumblr [6,35-37]. Previous studies have reported that between 25% and 33% of Facebook profiles of adolescents and young adults include references to depression symptoms consistent with the symptom criteria for a major depressive episode (MDE) [6].

Third, studies support links between displayed depression symptoms on SNSs and self-reported depression symptoms. One study found a positive association among young adults between posting displayed references to depression on Facebook and self-reporting depression symptoms [7]. Another study found that adults who used Facebook to post about health concerns were also more likely to have depression via a medical record review [38]. To date, these studies have focused on displayed depression symptoms in young adults or adults on SNSs.

Finally, previous literature has explored different approaches to examine the relationship between social media and depression, such as through studies examining the interaction between individual users and social media [39,40]. Other studies have examined social media language and compared it with the language present in medical records [41]. These studies complement approaches that examine social media directly and highlight the potential knowledge to be gained from understanding how depression is represented on social media and the user experience.

### The Role of Development

Adolescence is often the time when depression symptoms arise; thus, adolescents may be faced with these symptoms during a developmental stage in which their abstract thinking and ability to conceptualize the future is still developing [1-3]. As a result, adolescents may post their experiences on social media as an outlet for expression or to seek peer support. Developing and relying on peer support is a major task in adolescence. Given the social affordances of social media, these platforms may represent safe spaces in which adolescents feel comfortable expressing their new experiences. Alternatively, adolescents may be reluctant to share depression-related experiences on social media, as the fear of stigma among their peer groups may limit their comfort in expressing depression symptoms. As most studies to date of displayed depression symptoms on social media have focused on young adults, it remains unclear how and how often adolescents choose to represent depression symptoms on social media.

Young adulthood is a time in which youth achieve new levels of independence from parents and experience additional responsibilities for work or education. For young adults with depression, the developmental changes that occur in early adulthood can cause increased stressors [42]. Therefore, early adults may use social media to express these stressors to a known peer network where they can seek support. Previous studies have shown that young adult peers frequently see displayed depression symptoms by their peers and often interpret these displays as requests for support [34].

The developmental stage, which combines adolescence with young adulthood, has been proposed as a contributor to the symptom experiences of adolescents and young adults with depression [42]. A previous study argued that although the core experiences of depression were similar for adolescents and young adults, experiences related to stressors and depression triggers differed by developmental stage [43]. Other studies have found an improvement in depression symptoms in early adulthood compared with adolescence, suggesting that an increased level of experience, cognitive development, and psychosocial maturity may be key factors in improving an individual’s self-management of depression [44,45].

### Gaps in the Literature

At present, there are important gaps in the literature. First, this study is unique in its use of displayed depression symptoms on SNS samples at 2 developmentally distinct time points. There has been a call for studies on media about the adolescent developmental period [46]. Previous approaches using limited longitudinal study designs using SNSs have been successful. A previous study evaluated displayed references on SNSs regarding religion and sexual behavior over a one-year period [47]. A second study followed students during their first year of college to understand the emergence of alcohol displays on Facebook [48]. However, this study provides a unique contribution of examining 2 distinct time points, years apart, to understand differences by developmental period within a longitudinal cohort study.

Second, this study evaluates depression displays on SNSs at the symptom level. To date, most previous studies have focused on undifferentiated and aggregate displayed depression on SNSs. Depression symptoms can include sleep issues, which may be nonspecific to depression, and depressed mood, which is a more specific concern. The investigation of specific depression symptom displays on SNSs remains to be understudied. Furthermore, whether there are changes in displayed depression symptom frequency or types of depression symptoms displayed in adolescence compared with early adulthood remains to be unknown.

Third, studies provide conflicting findings regarding whether males or females are more likely to display depression references on SNSs. One previous study of college students found that 40% of females were depression symptom displayers (DSDs), compared with 25% of males [7]. Another study of college students found that females were more likely to display references to both stress and depression on SNSs [49]. Most of these studies have focused on college students, so it remains to be unclear how these results apply to adolescents or populations that are more general. A previous qualitative study focused on college students found that students perceived no differences in the frequency of seeing depression symptom disclosures on SNSs from male versus female peers [34].

To summarize, there is incomplete knowledge of depression displays by developmental stage, depression symptom displays, and gender, considering the unique time points of adolescence and young adulthood.

### Study Purpose

The aim of this content analysis study was to evaluate displayed depression symptoms on Facebook posts among a cohort of participants at 2 time points: adolescence and young adulthood. Our study objectives are to determine (1) the prevalence of displayed depression symptoms at 2 developmental time points: adolescence and young adulthood; (2) the most common depression symptoms displayed on Facebook; and (3) the prevalence of displayed depression symptoms by gender. As we conducted this study in the context of an ongoing longitudinal study, we also aim to illustrate our methodological process and its strengths and limitations so that future studies could consider such approaches in the context of their studies.

## Methods

### Overview

This study used a cohort of participants enrolled in the Developmental Pathways Project (DPP). This ongoing parent study was ideal for the purposes of this study for 3 reasons. First, the DPP study design was longitudinal and collected data from participants in adolescence (ages 17-18 years) and young adulthood (ages 21-22 years). Second, the study design of recruiting students in adolescence allowed us to leverage a more general population of adolescents, rather than focusing on college student populations, as in many previous studies. Third, recruitment was designed to oversample participants with early signs of depression, to increase the likelihood of having depression symptom displays present on SNSs.

The detailed screening and sampling procedures have been described previously [50,51]. Informed consent was obtained from adolescents and their parents at the start of the study. All study procedures were approved by the University of Washington Human Subjects Division.

### Participants

Recruitment began with mailing an introduction letter from the DPP principal investigators with a fact sheet for this study. This sheet explained that the purpose of the study was to learn about how young adults use social media. The participants were informed that we would be contacting them via email to request their Facebook profile name and URL to send a Facebook *friend request*. We created a Facebook profile for this study that participants would link their profile with for the duration of the study. We informed participants that if they accepted our *friend request* via Facebook, we would view their Facebook profile to get an idea of how often they used the profile and what they posted on it. They were assured that we would post no comments or public messages to their profile and that all information we gathered would be kept confidential. Participants were told that accepting the Facebook friend request served as their voluntary, informed consent to participate in the Facebook study. Participants received US $10 for participation.

One week after mailing the letter, we sent each eligible participant an email that contained the same letter and fact sheet and asked for their Facebook profile name and URL. For those who did not respond, we searched for their Facebook profile using their full name or email address. To verify that the participant’s Facebook profile belonged to the actual participant, one of the following data-matching criteria had to be met by finding the information on the Facebook profile and matching it to the information on file for that participant: their email, birthday, city and high school, city and high school graduation year, or high school and high school graduation year.

One week later, we sent friend requests to DPP participants who met the eligibility criteria for whom we had Facebook profile names. The friend requests included a Facebook message explaining that there was a new DPP-related study opportunity and that if they wished to participate, they would need to accept the friend request within 3 months. If we were unable to find the Facebook account, we sent a follow-up email requesting account information. If the participant had not accepted the friend request one week later, we sent a text message reminding them to check their email and Facebook accounts. If the participant still had not friended the Facebook account after 3 months, the friend request was rescinded.

### Depression Symptom Display Codebook Development

Using previous studies, we created a depression codebook containing keywords used to evaluate Facebook profiles and Twitter feeds for references to depression [6,35,36]. References to depression symptoms were defined using the symptoms outlined in the Diagnostic and Statistical Manual of Mental Disorders, Fourth Edition (DSM-IV) criteria for an MDE (Table 1) [52]. We chose the established clinical criteria as the basis of our research codebook to provide the most clinically relevant, objective framework for profile evaluation. The criteria for MDE based on the version of the DSM used in this study included depressed mood, loss of interest or pleasure in activities, weight or appetite changes, sleep problems such as insomnia or hypersomnia, slowing down of thought and movement (referred to as psychomotor retardation in the DSM-IV), energy loss, feelings of worthlessness or guilt, decreased concentration, or suicidal ideation [52]. Some of these criteria in the DSM-IV are further described as requiring observation by a physician to be considered valid, such as changes in appetite or weight. However, as this study involved evaluating self-disclosed feelings, thoughts, and experiences by participants, all symptoms were evaluated at face value, as reported by the participant.

**Table 1:** Depression symptom definitions applied to Facebook profile status updates.

DSM-IV^a^ criteria	Example key words or phrases	Exclusions
Depressed mood	Sad, empty, crying, tearful, alone, lonely, distressed, down (unless context clarifies otherwise)	“I had a bad day”: reference to a negative event without expressing emotion, FML (f*ck my life)
Decreased interest or pleasure in activities or anhedonia	Not having fun, don’t feel like doing anything, giving up, lack of purpose, not caring, not giving a “f*ck”	“I’m bored”: references to being bored or decreased interest in something specific such as a class or project
Changes in weight or appetite	No appetite, don’t feel like eating, can’t stop eating, eating everything in sight	“I ate too much McDonalds this weekend”: references to poor eating habits rather than change in appetite or references to single episodes of eating too much, such as at Thanksgiving
Insomnia or hypersomnia	Tired, exhausted, sleepy, need a nap, easily tired, not sleeping well, restless sleep, can’t sleep, can’t get to sleep, falling asleep in an unusual place, insomnia	“I took a nap after finals”: references to specific fatigue-related events
Agitation or slowing down of movement	Inability to sit still or feeling slow	“So excited for this concert that I can’t sit still!”: references to energy increases linked to specific events
Fatigue or loss of energy	Can’t get anything done, can’t get motivated due to fatigue	“I’m tapped out after that soccer game”: references to specific events or activity leading to low energy
Feelings of worthlessness or guilt	Feel guilty or worthless, “I am stupid,” “I’m not cool,” “I’m average,” “I’m crazy” or “I’m insane,” regretting something, being a failure, or failing	“I’m feeling so guilty after binge watching the whole fifth season”: references to single episodes of guilt linked to one-time activities
Difficulty concentrating or indecisiveness	Can’t study, can’t finish work, can’t concentrate because of emotion, can’t decide on something, don’t feel like deciding, can’t make up your mind, not knowing what to do	“I can’t concentrate on this homework because I am on Facebook”: references to not wanting to concentrate, or can’t concentrate because of activity
Recurrent thoughts of death	Thinking of ways to commit suicide, references to jumping, referencing death of self, thinking about death	—^b^

^a^DSM-IV: Diagnostic and Statistical Manual of Mental Disorders, Fourth Edition.

^b^Not available. No data fit this category.

Text-based Facebook displays were coded as depression references if they fit one of the described depression criteria based on keywords or synonyms. For example, one symptom of major depression is depressed mood; therefore, a status update stating “I feel depressed” would be coded as a depression symptom reference. Feeling down is often considered synonymous with feeling sad; therefore, a status update disclosing “I feel really down” would be coded as a depression symptom reference. Status updates or comments that referenced the common experience of having a bad day, such as “in a bad mood today because of my math exam,” would not meet the criteria for depression symptoms.

For this study, we began with the previously established codebook and engaged coders in testing and revising it for this study to ensure that symptom categories were well-developed and represented the current language on SNS profiles. A total of 4 rounds of pilot testing were conducted with participant profiles that were not included in the study data set. Modifications and revisions to the codebook were made to ensure that specific diagnostic criteria were represented clearly and as objectively as possible. Table 1 presents the revised codebook with examples of the symptom types.

### Depression Symptom Display Coding Procedure

#### Coding Depression Symptom Displays by Time Point

To evaluate depression symptom disclosures, coders reviewed each profile’s history of status updates and comments beginning one day before the date of interview at time 1 (ages 17-18 years) and time 2 (ages 21-22 years) and coded in reverse chronological order back to the same date, a year ago. Therefore, each period was tailored to the individual participant linked to the interview date but covered an identical range of time of 1 year. Each coding period was thus separated by 3 to 4 years.

#### Coding Depression Symptom Displays: Exclusions

For each social media post examined, if the post did not include any reference to depression symptoms, it was not coded. Status updates or comments that referenced a person other than the profile owner (ie, “Matt is sitting next to me in class and he looks bummed”) were not considered personal references and were excluded. Only text was evaluated in this study; thus, photos, links, and videos were excluded from the evaluation.

#### Coding Depression Symptom Displays: Process

For profiles in which a status update or comment disclosed a depression symptom, investigators categorized the type of depression symptom into one of the specific symptom categories. The unit of coding was a statement matching the depression symptoms. Therefore, if a post included multiple statements describing multiple symptoms, then each statement was counted as an individual depression symptom. For any displayed depression symptoms that met the criteria for representing suicidal ideation, researchers used an algorithm from previous studies to determine whether a response to that post was warranted [36]. Responses could include providing mental health resources or contacting participants to check if the concerning post was recent.

#### Interrater Agreement

Interrater agreement across depression symptom coding categories was compared across all the coders. The interrater agreement for depression symptom type ranged from a low of 88% for anhedonia to 98% for sleep concerns.

### Analysis

Qualitative assessment of the data was performed via content analysis. All coders went through a training phase of coding using pilot data not included in this study and began coding for this study once interrater agreement goals of >90% were met. When coding posts that met the criteria as a depression symptom, coders evaluated symptom types and placed them into appropriate symptom categories based on the codebook. Weekly meetings of all coders allowed the discussion of questionable codes and decisions were made by consensus.

For quantitative data, demographic variables and displayed depression references on Facebook were evaluated using descriptive statistics. We used Wilcoxon rank sum tests to test for associations between gender and the number of Facebook depression displays at each time point. Chi-square tests were used to test for an association between the presence of depression display and gender at each time point. All *P* values were two-sided, and *P*<.05 was used to indicate statistical significance. Statistical analyses were conducted using Stata 12 (StataCorp LP).

## Results

### Overview

This study was funded in October 2014 and received institutional review board approval from the University of Washington in February 2015. Participants from the larger DPP study were recruited for this study between February and April 2015. Retrospective data collection using Facebook profiles was conducted between April 2015 and August 2016. Statistical analyses and data interpretation were conducted subsequent to that time.

### Participants

Of 394 participants, 212 (53.8% of the DPP sample) participants were eligible for recruitment on the basis of the inclusion criteria. Among these 212 eligible DPP participants, 199 (93.9%) participants provided information about their Facebook profile, and 89 (41.9%) participants consented to participate in this study. A total of 78 out of 89 participants accepted our friend request and had data available at the time 2 data point.

At time 1, the sample of participants who had a Facebook profile as an adolescent included 48 participants aged between 17 and 18 years, of whom 48% (23/48) were male. The time 2 sample of participants who had a Facebook profile as a young adult included 78 participants, aged between 21 and 22 years, of whom 51% (40/78) were male.

### Depression Symptom Displays by Time Point

At time 1, 62% (48/78) of the participants had Facebook profiles and were included in the analyses. Of these 48 individuals whose Facebook profiles were coded, 27 (56%) displayed one or more depression symptoms on Facebook. We will refer to these participants as *DSDs*. The total number of depression displays over the year among all DSD profiles was 269. DSDs had an average of 5.3 (SD 9.2) depressive symptom displays and a median of 1 depressive symptom display in the one-year period. At time 1, the Facebook profile often represented status updates in the third-person language. A typical status update would read as “[Profile owner name] is....” Thus, many status updates were third person, such as “is feeling very sad.” Common types of language associated with these status updates included the use of all capital letters for emphasis (eg, “can’t stop EATING”) and unique spellings in shorthand (eg, “sleep d-prived”).

At time 2, all 78 participants had Facebook profiles and were included in the analyses. Of these 78 participants, 35 (45%) were DSDs. The total number of depression symptom displays by these DSDs at time 2 was 172. At time 2, DSDs had an average of 4.6 (SD 3.9) and a median of 3 displays. While by this point on Facebook, the third-person status update was no longer the norm, we saw the continued use of all capital letters for emphasis (eg, “just ate SO MUCH”) and seemingly purposeful misspellings, such as “I don’t wanna.”

### Depression Symptom Types by Time Point

Depression symptom displays were present across both time points (Table 2).

**Table 2:** Displayed depression symptom type on Facebook profiles by time point.

Depression symptom type	Time 1 (17-18 years; 2008-2010)		Time 2 (21-22 years; 2011-2014)	
	Posts (n=255), n (%)	Examples	Posts (n=167), n (%)	Examples
Depressed mood	36 (14.1)	“is kind of sad,” “is terrible, thanks for asking,” “is really, truly depressed”	32 (19.2)	“feeling the worst right now, just wanting to cry...,” “can’t depend on anyone, so sad”
Decreased interest or pleasure in activities or anhedonia	43 (16.9)	“doesn’t care anymore,” “too late to pull together the value of my life”	27 (16.1)	“and everything continues to suck...,” “what is this life? other than a slow and painful death”
Changes in weight or appetite	3 (1.2)	“fasting the rest of the week,” “can’t stop... EATING”	4 (2.4)	“craving every sort of food,” “another day of overeating”
Insomnia or hypersomnia	85 (33.3)	“5 naps in a day, phew,” “sleep d-pri-ved”	69 (41.3)	“just another night of insomnia,” “i don’t want to wake up”
Agitation or slowing down of movement	11 (4.3)	“so restless,” “feels paralyzed”	0 (0)	N/A^a^
Fatigue or loss of energy	3 (1.2)	“says get the heck up!” “feeling a bit listless...”	4 (2.4)	“don’t wanna do a thing,” “no motivation”
Feelings of worthlessness or guilt	34 (13.3)	“no one likes me,” “tired of not being good enough,” “wishing she was beautiful”	18 (10.8)	“officially a weekend loser... fail,” “ahhh i struggle with life”
Difficulty concentrating or indecisiveness	25 (9.8)	“NEEDS to learn how to focus!” “what am i doing with life...”	6 (3.6)	“don’t know how to feel...,” “so clueless and lost, i am”
Recurrent thoughts of death	15 (5.9)	“going to die,” “I am terminal,” “hard times call for drastic measures- got a gun,” “dad I’m still here, wishing I was dead with you.”	7 (4.2)	“have a party at my funeral,” “if I died tomorrow, what would you say to me,” “i wanna kill myself”

^a^N/A: not applicable.

At time 1, depression symptom displays included an average of 3.3 symptom types (SD 2.3; median 2) per participant. A common symptom type was sleep issues; 85 posts referenced sleep challenges, such as “5 naps in a day, phew” and “tired, I don’t want to wake up.” Another common type of depression symptom display was decreased interest or pleasure in activities (43 posts referenced this), such as “doesn’t care anymore” and “not having fun ever.”

At time 2, status updates referencing depression included an average of 2.4 (SD 1.3; median 2) depression symptoms among 9 possible symptom types. Similar to time 1, commonly displayed symptoms at time 2 included sleep issues (69/167, 41.3% of depression symptom posts), depressed mood (32/167, 19.2% of depression symptom posts), and decreased interest or pleasure (27/167, 16.2%). Table 2 shows examples of depression posts by symptoms.

### Prevalence of Displayed Depression Symptoms by Gender

Depression symptoms were observed in both females and males at both time points (Table 3).

At time 1, 52% (12/23) of males and 60% (15/25) of females displayed depression symptom references on Facebook; the prevalence estimates were not significantly different (*P*=.59). The median number of depression symptom displays for females and males was not significantly different (*P*=.28). The median number of displays for both genders was 1.

Across each of the 9 depression symptom categories except suicidal ideation, females consistently had a high proportion of displays; however, these differences were not statistically significant (*P*=.31 and *P*=.72 with Yates correction).

At time 2, displayed depression symptoms were present in 48% (19/40) of male profiles and 42% (16/38) of female profiles (*P*=.63); there were no significant differences by gender in the number of displayed depression symptoms (*P*=.94).

While comparing females and males across types of depression symptoms, males had higher rates of decreased interest or pleasure and loss of energy compared with females; however, these differences were not statistically significant (*P*=.11 and *P*=.40 with Yates correction).

**Table 3:** Facebook depression symptom displays by gender at time 1 and time 2.

Time point	Gender	Participant, n	Facebook depression symptom display, n (%)	Depression symptom displays on Facebook profiles	
				Value, mean (SD)	Value, median (range)
**Time 1 (n=48)**					
	Males	23	12 (52)	2.7 (5.7)	1 (0-25)
	Females	25	15 (60)	7.6 (11.2)	1 (0-33)
**Time 2 (n=78)**					
	Males	40	19 (48)	1.6 (2.8)	0 (0-11)
	Females	38	16 (42)	2.4 (3.9)	0 (0-15)

## Discussion

### Principal Findings

This study applied content analysis to evaluate displayed depression symptoms on Facebook within an ongoing longitudinal cohort study of adolescents. The findings contribute to the state of the science regarding how symptoms of depression are displayed on SNSs at 2 time points across a critical developmental period of emerging adulthood: by time, symptom, and gender. We identified that DSDs were common at both time points, with a variation in prevalence and symptom type among adolescents and young adults. We did not identify any gender differences in the prevalence of depression symptom displays at either time point.

### Types and Timing of Displayed Depression Symptoms on Facebook

The first finding is the high prevalence of displayed depression symptoms at both time 1 and time 2. This finding is different from that previously reported in the literature. Previous studies have reported that the prevalence of displays of depression symptoms on Facebook ranges between 25% and 33% [6,7]. It is likely that this study sample, which was oversampled for early signs of depression as part of the prospective cohort design, was a major contributing factor to the higher prevalence of displayed depression symptoms in this study. However, it is also important to note that most previous studies have focused on college students. This study focused on adolescents, not all of whom were in college at the young adult time point. As this is the first study to examine displayed depression disclosures by a population of adolescents, our study findings support the importance of future work focused on the adolescent population to better understand how displayed depression symptoms may present opportunities to better understand, detect, or provide early intervention for depression.

Furthermore, we found that 53% (41/78) of our participants chose to represent depression symptom displays on Facebook as adolescents and about 44% (34/78) chose to represent depression symptom displays on Facebook as young adults. Another interesting finding is that at time 1, our 38 participants generated 269 depression symptom displays, whereas at time 2, our larger number of 78 participants posted a smaller number of 172 depression displays. These findings align with previous longitudinal studies of the emerging adolescence period, and one study found decreases in depression symptoms over 7 years between adolescence and early adulthood [44]. These results support early adulthood as a time in which many early adults experience improved psychological well-being and psychosocial maturity, which may manifest via decreased depression disclosures on social media.

A second finding was that both adolescents and young adults represented their depression symptoms using all 9 symptom types, including mood symptoms, physical symptoms, such as sleep, and even suicidal ideation. Furthermore, participants expressed these symptoms in informal ways, such as slang, shortened text, and all capitals for emphasis. This language may be more commonly attributed to the adolescent developmental stage but was observed across both time points in our study. In addition, both adolescents and young adults were willing to share information that may be seen as quite deep and personal information about how they were feeling, including clear descriptions of depression symptoms, such as hopelessness. We also found descriptions of poor self-worth, sleep challenges, and appetite changes. It is possible that having a network of peers on SNSs allows emerging adults to share developmental concerns around self-worth, sleep difficulties, and changing appetites that are fundamental to the developmental transition from adolescence to young adulthood.

The rich descriptions that emerging adults were willing to share may be startling as representations of publicly shared text. However, these disclosures may reflect an emerging cultural norm around what types of content are acceptable to share, both in a web-based public format and among a new generation of emerging adults who may view mental health as less stigmatizing compared with previous generations. It is also possible that some of these disclosures were not perceived by participants as depression symptoms, such as disclosures related to sleep problems or weight changes. Previous studies have illustrated that emerging adults often receive peer support for these disclosures, which may include social media comments in response, or offers to get together offline [6,53]. Thus, some adolescents and young adults may display depression symptoms in an effort to reach out for help or support from their peer network. Peers may be the first to observe and respond to depression symptom disclosures, and symptoms suggesting hopelessness or suicidal ideation may be the most critical for peer response. Future studies exploring the motivations and mechanisms of peer support in response to displayed depression symptoms are warranted.

### No Differences in Depression Symptom Displays on SNSs by Gender

A third finding was that no differences in the prevalence of displays or types of symptoms displayed between females and males were found in this study. This finding may seem surprising, given that the literature suggests that females more often display personal or relational content and mention more psychological and social processes, such as emotions and feelings [3,6]. There are several possible explanations for the absence of gender differences in our study. It is possible that some aspects of the initial DPP study design, such as the sampling procedures for the original DPP study, might have influenced SNS disclosure patterns for both females and males in ways that we cannot understand. It is also possible that SNSs allow males a safe or culturally appropriate place to share depression symptoms or experiences. Previous research focused on early computer use communication found that human-computer interaction can lead to increased self-disclosure and less inhibited personal expression [54,55]. It is possible that this expression in males allows them the opportunity to express emotions in ways that may be more intimidating in offline communication. Thus, SNSs may be a venue in which males feel comfortable expressing depression symptoms.

It is interesting to consider another perspective that it is possible that our coding approach may have underestimated the frequency of male depression disclosure. It has been suggested that males are more likely to endorse other symptoms of distress, such as aggression, substance abuse, and risk-taking behavior, whereas women are more likely to endorse symptoms of sadness, stress, irritability, sleep problems, and loss of interest [56]. Our coding approach was based on the DSM-IV, and the symptoms of distress commonly noted by males were not among the criteria. However, the DSM-IV criteria does include the symptoms most commonly reported by women. Therefore, it is possible that some males’ disclosures related to underlying depression may have been missed based on our coding criteria, leading to an underestimation of mental health issues of males displayed on social media.

### Limitations

Our study had several limitations. First, although the overall sample size of the cohort study from which we recruited for this study was large, the number of participants who met our inclusion criteria and were recruited was relatively small. The smaller sample and cohort study design allowed us to thoroughly evaluate participants’ displayed symptoms at 2 time points, yielding rich participant-generated data that allowed both quantitative and qualitative analyses. However, ongoing studies that integrate social media approaches as additional data collection may improve their participant numbers through approaches such as phone calls to participants or increased stipends. Another limitation of our study recruitment approach is potential sampling bias, as the participants who consented may have had different Facebook display patterns compared with those who declined participation. Furthermore, this study’s purpose was to evaluate user-generated text describing depression symptoms applying the DSM-IV criteria, which are also in writing and not represented via images. Thus, multimedia content such as photos, videos, or links to articles was not evaluated, although there are no current criteria or standards defined to evaluate such content. We evaluated all disclosures through the lens of the DSM-IV but did not factor in additional requirements for some criteria to be observed by a clinician, given our study’s focus on social media displays by participants. In taking all public web-based disclosures at face value, we cannot make claims about the motivation or context for participants’ posts. Another limitation is that we evaluated displayed depression symptoms on only one site, Facebook. As noted repeatedly in this paper, Facebook as a platform is not a static entity but is dynamic and changes over time. These changes were well illustrated within our data; at time 1, status updates were in the third-person language, and by time 2, this was no longer the case. Therefore, aspects or patterns of display at the earlier or later data collection times in our study may have changed. This is a limitation of any social media study and emphasizes the importance of reflecting on overall aspects of communication and culture around social media, such as through an affordance approach, rather than particular nuances of a single platform. Finally, because this study was retrospective within an existing cohort, data are not recent and may not reflect current trends in depression symptom displays on social media.

### Conclusions and Implications

Despite these limitations, our study provides novel findings that contribute toward understanding how adolescents and young adults represent depression symptoms on Facebook during the critical developmental period of emerging adulthood. An important research implication of our study findings is that our findings can contribute to other studies in this important area. First, our study may inform text mining or big data studies of depression disclosure. It is important to note that many of the depression symptoms coded in this study that met the criteria within our codebook would not likely be identified through big data approaches tied to keywords. Adolescents used slang and deliberate misspellings, such as “don’t wanna do anything,” to represent constructs such as anhedonia. Studies using keywords or big data approaches to mine text-based social media data may find it useful to consider unexpected yet common word combinations to identify content that may signify depression symptoms. A previous systematic review noted that assembling large data sets of mental health symptoms on social media is problematic because of limitations and biases with data collection methods [57]. Combining *thick data*, such as what was collected in this study through human coders, with big data approaches using automated detection may allow for a broader yet more nuanced understanding of depression.

A second implication for future studies is to use our findings to design future studies more narrowly focused on specific or critical depression symptoms. Our study took the approach of understanding the ways in which all types of depression symptoms may be represented on Facebook. One finding was that depression symptoms around sleep concerns were common, although this particular symptom type could be interpreted as nonspecific for depression. Thus, future studies may elect to narrow the focus on sleep-related coding to only code posts that specifically mention sleep issues and depression. Alternatively, future studies could consider focusing on coding symptoms that are more specific to depression, such as depressed mood.

Furthermore, our study provides methodological contributions on how existing studies may incorporate SNS evaluations into the study design. We found that using an existing study sample from a longitudinal cohort study was feasible and provided a novel approach to the evaluation of depression symptom displays on Facebook. We found that from our larger longitudinal sample, very few participants had data at the 2 time points evaluated. This finding may be applicable to other studies, as social media patterns and platforms change over time. Finally, given the frequency of depression symptom displays, SNSs may be an innovative avenue for raising self-awareness and combating stigma surrounding mental health conditions. As social media continues to evolve, further studies to evaluate these types of displays over time or across platforms are warranted.

## Abbreviations

DPP: Developmental Pathways Project
DSD: depression symptom displayer
DSM-IV: Diagnostic and Statistical Manual of Mental Disorders, Fourth Edition
MDE: major depressive episode
SNS: social networking site
